# Increased incidence trend of low**-**grade and high-grade neuroendocrine neoplasms

**DOI:** 10.1007/s12020-017-1273-x

**Published:** 2017-03-16

**Authors:** Emanuele Leoncini, Paolo Boffetta, Michail Shafir, Katina Aleksovska, Stefania Boccia, Guido Rindi

**Affiliations:** 1 Section of Hygiene, Institute of Public Health, Rome, 00168 Italy; 2 Tisch Cancer Institute, New York, NY 10029 USA; 3 Departments of Surgery and Neoplastic Diseases, Icahn School of Medicine at Mount Sinai, 1 Gustave L. Levy Pl, New York, NY 10029 USA; 4 Institute of Anatomic Pathology, Università Cattolica del Sacro Cuore, Largo A. Gemelli, 8, Rome, 00168 Italy; 5 European NeuroEndocrine Tumor Society (ENETS) Center of Excellence, Rome, Italy

**Keywords:** Neuroendocrine, Cancer, Low-grade, High-grade, Incidence

## Abstract

**Purpose:**

The incidence of neuroendocrine neoplasms is increasing. This work aimed at: (i) establishing worldwide incidence trend of low-grade neuroendocrine neoplasms; (ii) defining the incidence and temporal trend of high-grade neuroendocrine neoplasms in USA utilizing the Surveillance Epidemiology and End Results database; (iii) comparing trends for low-grade vs. high-grade neuroendocrine neoplasms.

**Methods:**

We conducted a literature search on MEDLINE and Scopus databases and incidence trends were plotted for 1973-2012. The Surveillance Epidemiology and End Results database was used to identify incidence rates in USA for 1973-2012. Incidence rates were stratified according to histological grade, gender and ethnicity. Trends were summarized as annual percent change and corresponding 95% confidence interval.

**Results:**

11 studies were identified involving 72,048 cases; neuroendocrine neoplasm incidence rates increased over time in all countries for all sites, except for appendix. In Surveillance Epidemiology and End Results low-grade neuroendocrine neoplasm incidence rate increased from 1.09 in 1973 to 3.51 per 100,000 in 2012. During this interval, high-grade neuroendocrine neoplasm incidence rate increased from 2.54 to 10.52 per 100,000. African Americans had the highest rates of digestive neuroendocrine neoplasms with male prevalence in high-grade.

**Conclusions:**

Our data indicate an increase in the incidence of neuroendocrine neoplasms as a worldwide phenomenon, affecting most anatomical sites and involving both low-grade and high-grade neoplasms.

## Introduction

Neuroendocrine define those neoplasms exclusively made by cells with a neuroendocrine phenotype, i.e., expressing markers of neuroendocrine differentiation like chromogranin A, synaptophysin, neuron specific enolase and others including hormones. As such, neuroendocrine neoplasms (NENs) may develop at any anatomical site [1]. The present paper focuses on NENs of the gastroenteropancreatic (GEP) tract. The current World Health Organization (WHO) classification of GEP NENs defines neuroendocrine tumor (NET) as well differentiated low to intermediate grade, and neuroendocrine carcinomas (NEC) as high grade neoplasms, poorly differentiated in phenotype [2]. Recent evidences also identified well differentiated NENs of high grade (for review and definition *see* [3]), now endorsed by the American Joint Cancer Committee Cancer Staging Manual [4, 5] and heralding a yet-to-come WHO classification change.

Global incidence of GEP NENs appears to be increasing. In the last four decades, incremental trends were reported in various populations for NENs overall and for specific primary sites [6–16]. Data were obtained from different population-based registries, which varied in completeness and time periods. Seminal papers were generated from the Surveillance, Epidemiology, and End Results (SEER) program of the US collecting cancer information since 1973, probably one of the most complete cancer registry publicly available in western countries [9, 17].

Published investigations focused broadly on NENs and were generated utilizing general “neuroendocrine” ICD-O codes potentially mixing cancers with different biology. Available data are thus difficult to interpret, and are commonly intended as referring to NENs of low to intermediate grade (from now on low-grade NENs), either defined as carcinoids, atypical carcinoids or, more recently, neuroendocrine tumors (NETs). Limited epidemiological data is available for poorly differentiated, high-grade NENs (from now on high-grade NENs) [18, 19].

Aims of this work were: (i) establishing worldwide trends in the incidence of low-grade NENs; (ii) defining the incidence and temporal trend of high-grade NENs in USA utilizing the Surveillance Epidemiology and End Results (SEER) database; (iii) comparing trends for low- vs. high-grade NENs.

## Materials and methods

### Published studies data analysis

#### Search strategy

We conducted a systematic review according to PRISMA guidelines [20] aiming at identifying studies on NEN incidence. We searched MEDLINE and Scopus with the following keywords: “neuroendocrine”, “carcinoid”, “epidemiology”, “trend” and “incidence”. The search was limited to English studies on human subjects till October 1, 2015. References of included articles were screened for any additional eligible study.

#### Inclusion and exclusion criteria

Inclusion criteria required that the study: (i) reported incidence estimates for NENs overall or for site-specific NENs; (ii) reported incidence estimates for at least 10-years with at least two time points; (iii) used data series from population-based surveillance systems. If articles reported data on overlapping regions or time intervals, the study with the most recent information was included. Studies reporting NEN incidence estimates in USA were excluded as we generated results for this country based on the most recent data available from the SEER database.

#### Data extraction

Titles and abstracts of articles obtained from the literature search were reviewed for eligibility by two authors (EL and KA). Papers successfully meeting the inclusion criteria were selected for data extraction. The investigators independently extracted the following information: first author’s name, publication year, study location, study period, cancer site (including topographical and morphological codes), number of cases, incidence rates, and standard population used for adjustment. We extracted data according to sex or racial/ethnic group, whenever available.

#### Summarization of data

International trends in incidence were plotted for all NENs combined and for most common primary sites for the period 1973–2012. Measures of incidence presented are those reported in the individual studies. Any measure also depends on the standard population used for the adjustment. When studies reported incidence rates separately for men and women, an average incidence rate was computed.

#### NEN incidence in the United States

We used the SEER database to identify NEN incidence rates in the United States [21]. The SEER program, started in 1973, currently registers cancer incidence and subsequent cause-specific mortality in 30% US population.

In order to provide comparable data with published incidence data, but also to be as much as possible consistent with the current classification systems for pure NENs, records were selected based on the following ICD-O-3 (International Classification of Diseases for Oncology) morphological codes: M8150/3 (Islet cell carcinoma), M8151/3 (insulinoma), M8152/3 (glucagonoma), M8153/3 (gastrinoma), M8155/3 (vipoma), M8156/3 (somatostatinoma), M8240/3 (carcinoid tumor, not appendix), M8241/3 (carcinoid tumor, argentaffin), M8242/3 (enterochromaffinlike-cell tumor), M8246/3 (neuroendocrine carcinoma), M8249/3 (atypical carcinoid).

Low-grade NENs were selected based on the following ICD-O-3 codes: M8150/3, M8151/3, M8152/3, M8153/3, M8155/3, M8156/3, M8240/3, M8241/3, M8242/3, M8249/3; whereas high-grade NENs were selected based on the following codes: M8013/3 (large cell neuroendocrine carcinoma), M8041/3 (small cell carcinoma), M8246/3 (neuroendocrine carcinoma). Codes identifying cancer types of mixed neuroendocrine/non-neuroendocrine phenotype (e.g., goblet cell carcinoid, mixed adeno-neuroendocrine carcinoma) were excluded.

### Statistical analysis

Age-adjusted incidence rates of NENs for the most common primary sites (lung and bronchus, stomach, pancreas, small intestine, colon, appendix, rectum) and for all sites combined for the 40 years period 1973–2012, were reported. Incidence data were obtained from the SEER 9 registries for years 1973–1991, from the SEER 13 registries for years 1992–1999 and from the SEER 18 registries for the years 2000–2012.

Incidence rates according to histological classification of malignancy grade and by gender and by ethnicity (white, black, Asian Pacific/Islander) for 2000 through 2012 were collected from the SEER 18 registries were reported to further assess whether overall changes in incidence rates were uniform or more marked for specific groups. All rates are expressed per 100,000 persons and age-adjusted according to the 2000 US standard population.

Trends for the period 2000–2012 were summarized with the annual percent change (APC) (Joinpoint Regression Program, Version 4.3.1.0 - April 2016; Statistical Methodology and Applications Branch, Surveillance Research Program, National Cancer Institute) and corresponding 95% confidence interval (CI) [22]. A sensitivity analysis of trends according to histological grade restricted to the SEER 9 registries with complete data during the period 1975–2012, was done.

Male-to-female (M:F) ratios, based on the most recently available incidence data from the 2010–2012 SEER, were also computed. Stata software (StataCorp. 2013. Stata Statistical Software: Release 13. College Station, TX: StataCorp LP) was used for data management and to produce graphs.

## Results

### Review of worldwide incidence of NEN

**Fig. 1:**
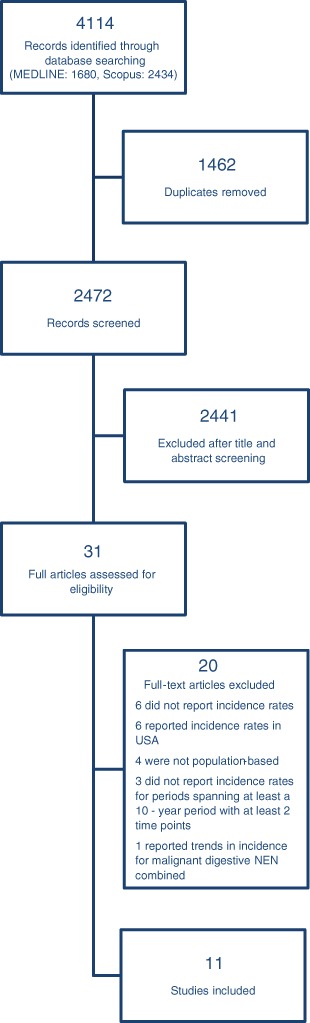
Flow diagram of study selection

**Fig. 2:**
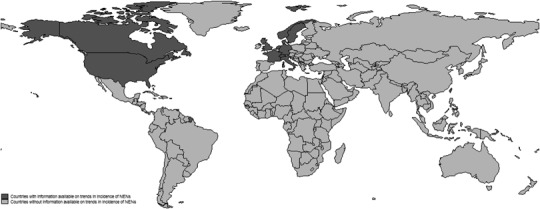
Countries with information available on trends in incidence of NENs

The results of the bibliographic search are summarized in Fig. 1. A total of 4114 articles were considered. After excluding duplicates, reviews and studies unrelated to the search topic, we assessed for eligibility of 31 full-text articles. Of these, 20 articles were excluded after full review. Table 1 shows the main characteristics of the studies included and the SEER Program. The 11 studies were published between 2000 and 2015 and involved a total of 72,048 cases. Nine were conducted in Europe [6–12, 15], one in Canada [16], and one in Taiwan [13] (Fig. 2). In five countries (Denmark, England, The Netherlands, Norway, Taiwan), the incidence data covered the entire national population, while in the other six countries, covered 1% to 30% of the population.

**Table 1:** Summary of the studies reporting NEN incidence and the SEER Program of the National Cancer Institute

First author, publication year	Study period	Country	Study area	Total cases	Incidence rates, type of adjustment	Subsites of NEN incidence
North America						
SEER [present series]	1973–2012	USA	1973–1991: 9 Registries, 1992–1999: 13 Registries, 2000–2012: 18 Registries	na	Age-standardized, 2000 US Standard Population	Overall, lung, stomach, pancreas, small intestine, colon, appendix, rectum
Hallet, 2015	1994–2009	Canada	Ontario	5619	Crude	Overall, lung, stomach, pancreas, small intestine, colon, appendix, rectum
Europe						
Korse, 2013	1990–2010	Netherlands	Nationwide	47,800	Age-standardized, European Standard Population	Overall, lung, stomach, pancreas, small intestine, appendix, rectum
Scherubl, 2013	1976–2006	Germany	Mecklenburg-Western Pomerania, Saxony, Brandenburg or Thuringi	2821	Age-standardized, Population of Germany in 1987	Stomach, pancreas, small intestine, colon, appendix, rectum
Caldarella, 2011	1985–2005	Italy	Firenze and Prato	455	Age-standardized, 2000 European Standard Population	Overall
Ellis, 2011	1971–2006	England	Nationwide	10,324	Crude	Stomach, small intestine, colon, appendix, rectum
Landerholm, 2010	1960–2005	Sweden	Jonkoping County	145	Age-standardized, Population of Sweden in 2005	Small intestine
Hauso, 2008	1993–2004	Norway	Nationwide	2030	Age-standardized, 2000 US Standard Population	Overall, lung, stomach, pancreas, small intestine, colon, appendix, rectum
Lepage, 2006	1976–2001	France	Burgundy	102	Age-standardized, World standard population	Small intestine
Skuladottir, 2002	1978–1997	Denmark	Nationwide	347	Age-standardized, Population of Denmark in 1980	Lung
Levi, 2000	1974–1997	Switzerland	Vaud	218	Age-standardized, World Standard Population	Overall, lung, stomach, small intestine
Asia						
Tsai, 2013	1996–2008	Taiwan	Nationwide	2187	Age-standardized, 2000 US Standard Population	Overall, lung, stomach, small intestine, pancreas, rectum

*NEN* neuroendocrine neoplasm, *ICD-O* International Classification of Disease for Oncology, *SEER* Surveillance, Epidemiology, and End Results

Seven studies reporting details on ICD-O codes included the ambiguous neuroendocrine carcinoma (M8246/3; ambiguous since could have comprised both low-grade and high-grade NENs), six included codes of mixed neuroendocrine–non neuroendocrine cancer (e.g. M8243 goblet cell carcinoid), and two included the high-grade large cell neuroendocrine carcinoma (Table 2). Thus, the published data were not fully comparable and homogenous for neuroendocrine cancer types collected, either non-purely neuroendocrine and/or of different grades.

**Table 2:** NEN-related ICD-O codes used in SEER database extraction and in 11 studies included in the systematic review

	USA [present series], SEER, 2015	SWITZERLAND, Levi, 2000	DENMARK, Skuladottir, 2002	FRANCE, Lepage, 2006	NORWAY, Hauso, 2008	USA, Hauso, 2008	SWEDEN, Landerholm, 2010	UNITED KINGDOM, Ellis, 2010	ITALY, Caldarella, 2011	GERMANY, Scherubl, 2013	TAIWAN, Tsai, 2013	NETHERLANDS, Korse, 2014	Canada, Hallet, 2015
M8013/3 Large cell neuroendocrine carcinoma (§)		Not reported	Not reported	Not reported		●	Not reported				●		
M8041/3 Small cell carcinoma												●(#)	
M8150/3 Islet cell carcinoma	●								●	●		●	●
M8151/3 Insulinoma	●								●	●		●	●
M8152/3 Glucagonoma	●								●	●		●	●
M8153/3 Gastrinoma	●								●	●		●	●
M8154 Mixed islet cell and exocrine adenocarcinoma									●				●
M8155/3 Vipoma	●								●	●		●	●
M8156/3 Somatostatinoma	●								●	●		●	●
M8157 Enteroglucagonoma									●	●			●
M8240/1 Carcinoid tumor, appendix					●	●		●	●	●	●	●	●
M8240/3 Carcinoid tumor, not appendix	●				●	●		●	●	●	●	●	●
M8241/3 Carcinoid tumor, argentaffin	●				●	●		●	●	●	●	●	●
M8242/3 Enterochromaffinlike-cell tumor (§)	●							●	●	●	●	●	●
M8243 Goblet cell carcinoid					●	●		●	●	●	●		●
M8244 Composite carcinoid					●	●		●	●	●	●		
M8245 Adenocarcinoid tumor					●	●			●	●	●		●
M8246/3 Neuroendocrine carcinoma	●				●	●		●	●	●	●	●	●
M8248/3 Apudoma												●	
M8249/3 Atypical carcinoid (§)	●					●			●	●	●	●	●
M8574/3 Adenocarcinoma with neuroendocrine differentiation (§)						●				●	●		
ICD-O edition	ICD-O-3	ICD-O-1	ICD-O-1	ICD-O-2	ICD-O-2	ICD-O-2 and 3	Not reported	ICD-O-1 and 2	ICD-O-3	ICD-O-3	ICD-O-Field trial and 3	ICD-O-1,2 and 3	ICD-O-3

*NEN* neuroendocrine neoplasm, *ICD-O* International Classification of Disease for Oncology, *SEER* Surveillance, Epidemiology, and End Results

(§) New histology codes (ICD-O-3) for NETs, in use since 2001.

(#) In the present study we report the trends in incidence of NEN in The Netherlands excluding small cell neuroendocrine carcinoma (G3-SCNEC) to make data comparable with other studies

### NEN incidence in the United States

**Fig. 3:**
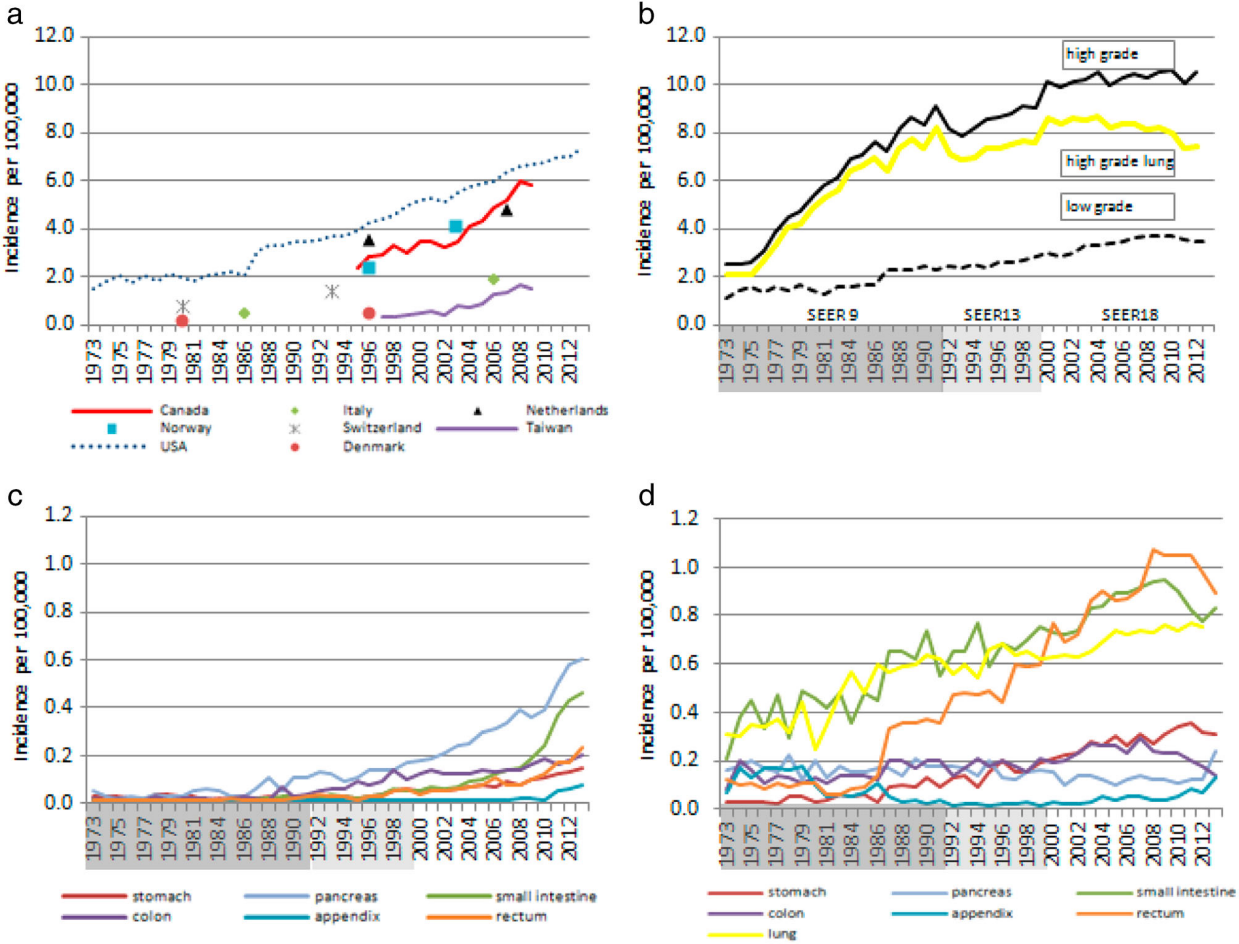
**a** International incidence of NENs overall per 100,000 persons; **b** Incidence of high-grade lung NEN, and low-grade and high-grade NENs per 100,000 persons in the United States, 1973–2012; **c** Incidence of low-grade NENs per 100,000 persons by primary site, in the United States, 1973–2012; **d** Incidence of high-grade NENs per 100,000 persons by primary site, in the United States, 1973–2012

Trends in incidence for NENs overall and for common primary sites are shown in Fig. 3a and detailed in Table 3. Incidence data of all NENs combined were available for seven countries. In USA and EU countries for which trend data was available, the incidence of NENs increased steadily. The incidence also increased in Taiwan (from 0.30 in 1996 to 1.51 in 2008), where it was relatively low compared to other countries. In the United States, NEN incidence increased from 1.52 to 7.41 cases per 100,000 from 1973 to 2012; this represented a 4.88-fold increase.

**Table 3:** NEN incidence per 100,000 by country and tumor site

		NEN incidence per 100,000								All sites average increase (cases per 100,000 per year)
Country	Year	Lung	Stomach	Pancreas	Small intestine	Colon	Appendix	Rectum	All sites	
North America										
USA	1973	0.31	0.03	0.16	0.21	0.08	0.07	0.12	1.52	0.16
	2012	1.61	0.45	0.82	1.28	0.30	0.22	1.10	7.41	
Canada	1994	0.83	0.07	0.10	0.42	na	na	0.22	2.46	0.23
	2009	1.28	0.29	0.60	1.01	na	na	0.96	5.86	
Europe										
The Netherlands	1990–00	0.68	0.15	0.19	0.32	0.13	0.58	0.19	3.57	0.11
	2001–10	1.21	0.19	0.32	0.47	0.19	0.59	0.29	4.76	
Germany	1976–78	na	0.01	0.06	0.13	0.05	0.28	0.01	na	nc
	2004–06	na	0.25	0.25	0.52	0.24	0.35	0.25	na	
Italy	1985	na	na	na	na	na	0.00	na	0.50	0.07
	2005	na	na	na	na	na	0.30	na	1.90	
England	1971–78	na	0.01	na	0.12	0.18	0.04	0.01	na	nc
	2000–06	na	0.16	na	0.39	0.17	0.50	0.11	na	
Sweden	1960–75	na	na	na	0.58	na	na	na	na	nc
	1991–05	na	na	na	1.33	na	na	na	na	
Norway	1993–97	0.49	0.15	0.15	0.60	0.19	0.10	0.22	2.35	0.24
	2000–04	0.90	0.20	0.30	1.01	0.33	0.23	0.25	4.06	
France	1976	na	na	na	0.07	na	na	na	na	nc
	2001	na	na	na	0.21	na	na	na	na	
Denmark	1978	0.20	na	na	na	na	na	na	na	nc
	1997	0.49	na	na	na	na	na	na	na	
Switzerland	1974–85	0.33	0.01	na	0.16	na	na	na	0.77	0.05
	1986–97	0.47	0.05	na	0.44	na	na	na	1.39	
Asia										
Taiwan	1996	0.05	0.02	0.02	0.03	0.02	na	0.07	0.30	0.11
	2008	0.27	0.13	0.13	0.06	0.09	na	0.38	1.51	

*na* not available, *nc* not computable

Incidence rates of all site-specific NENs increased over time in all countries for all sites, except for appendix (supplementary Fig. 1). Rates of appendix NENs have been increasing over time in England and Norway, while remained stable in Germany, the Netherlands and the United States.

### NEN incidence in the United States by grade

The age-adjusted incidence rates of NENs according to histological grade from 1973 to 2012 are illustrated in Fig. 3b–d. The overall incidence rate of low-grade NENs increased from 1.09 per 100,000 in 1973 to 3.51 in 2012 (Fig. 3b); this represented a 3.2-fold increase. During the same interval, the overall incidence rate of high-grade NENs increased from 2.54 per 100,000 to 10.52 (Fig. 3b); this represented a 4.1-fold increase.

During 2000–2012, the incidence rate of low-grade NENs (Fig. 3c and supplementary Fig. 2) increased in the lung (0.63 per 100,000 in 1973 vs 0.75 per 100,000 in 2012; APC: 1.74, 95% CI: 1.21, 2.27), stomach (0.22 per 100,000 in 1973 vs 0.31 per 100,000 in 2012; APC: 3.03, 95% CI: 1.65, 4.43), appendix (0.02 per 100,000 in 1973 vs 0.13 per 100,000 in 2012; APC: 12.07, 95% CI: 6.86, 17.54), and rectum (0.69 per 100,000 in 1973 vs 0.89 per 100,000 in 2012; APC: 2.52, 95% CI: 0.90, 4.17). By converse, the incidence rate of pancreas and small intestine tumors remained stable, while for colon it decreased (0.20 per 100,000 in 1973 vs 0.14 per 100,000 in 2012; APC: −2.65, 95% CI: −5.26, −0.03). In 2012, the highest incidence rate of low-grade NENs was observed for rectum (0.89 per 100,000), followed by the small intestine (0.83 per 100,000), and lung (0.75 per 100,000). Overall, the incidence of digestive low-grade NENs was 2.54 per 100,000.

During 2000–2012, the incidence rates of high-grade NENs (Fig. 3d and supplementary Fig. 2), increased over time for all sites, except for lung, for which a decreasing trend was observed (8.62 per 100,000 in 1973 vs 7.47 per 100,000 in 2012; APC: −1.14, 95% CI: −1.63, −0.64) (Fig. 3b and supplementary Fig. 2). During the same period, the small intestine showed the largest APC (0.07 per 100,000 in 1973 vs 0.46 per 100,000 in 2012; APC 20.68, 95% CI: 17.8, 23.7), while the colon showed the lowest APC (0.14 per 100,000 in 1973 vs 0.20 per 100,000 in 2012; APC 4.09, 95% CI: 2.56, 5.64) (Fig. 3d and supplementary Fig. 2). Lung cancer was the most frequently diagnosed high-grade NEN (7.47 per 100,000), accounting for 71% of the total new high-grade NEN cases in 2012. Overall the incidence of digestive high-grade NENs was 1.72 per 100,000.

Incidence rates of NENs from 2000 to 2012 according to grade by gender are presented overall and by anatomical site in Supplementary Figs. 3 and 4. Incidence rates of low-grade and high-grade NENs demonstrated a similar pattern in both males and females. When taking into account all the NEN cases over the last 3 years, the overall M:F ratio was 0.9 and 1.2 for low-grade and high-grade NENs, respectively.

Incidence rates of NENs from 2000 to 2012 according to grade for three ethnic populations are presented overall and by anatomical site in Supplementary Figs. 5 and 6. In the period 2010–2012, within the low-grade NENs, African Americans showed the highest overall incidence rate (5.57 per 100,000), followed by whites (3.28 per 100,000), and Asians/Pacific Islanders (2.21 per 100,000). In the same period, within the high-grade NENs, whites had the highest overall incidence rate (11.02 per 100,000), followed by African Americans (10.56 per 100,000), and Asians/Pacific Islanders (4.75 per 100,000).

Sensitivity analyses restricted to SEER 9 registries with complete data (period 1975–2012) revealed incidence trends for NEN according to histological grade, comparable to those obtained from different combinations of SEER registries in terms of both magnitude and statistical significance (data not shown).

## Discussion

This work aimed at defining the current pattern and trend in the incidence of NENs worldwide, and at analyzing the SEER database for more detailed results. Based on the codes here utilized, only pure neuroendocrine cancers were investigated. Additionally, of the recently recognized high grade NEN types (G3 NET and NEC), the present investigation applies to NEC only [3, 23–28]. Our data indicate that (i) the reported incremental trend observed for low-grade NENs is confirmed as a worldwide phenomenon, (ii) that this trend occurs at most anatomical sites and (iii) it is paralleled by an increase in the incidence of high-grade NENs.

The worldwide incremental trend for NENs is here confirmed at most anatomical sites investigated. No explanation for this phenomenon is apparent. A mix of better understanding of biology and clinical features of NEN and better diagnosis was offered as the most likely interpretation [17]. Though this could be the case, the link to potential NEN-specific promoting agents cannot be excluded. As an example and in line with this hypothesis the widespread access to proton-pump inhibitors has been proposed as possible risk factor for gastric NEN development [29]. Recent data from Norway however suggest a true increase of NENs [18, 19].

The observed rapid increases in incidence rates associated with significant variations in national NEN incidence rates. Our data indicate that geographic variations remained stable over time, the highest incidence rates observed in North America and relatively low rates in Taiwan. These variations may well reflect heterogeneity in disease classification since classification changes occurred in recent years [2]. Nonetheless little is known of environmental, ethnic/genetic or other risk/predisposing factors that could be involved [30].

Here we report also the age-adjusted incidence rates of NENs according to histological grade in US. Our data show that the incidence of high-grade NENs is overall significantly higher when compared to low-grade NENs (10.52 vs. 3.51 per 100,000 in 2012). However, this reflects the very high incidence observed for lung, as compared to digestive NENs (7.47 vs. 1.72 per 100,000 in 2012). These site-dependent features further confirm significant site-specific differences.

In the period 2000–2012, the incidence rates of high-grade NENs in SEER increased over time for all sites, except for lung, where a decreasing trend was observed. This latter observation may well reflect the change in smoking habits in US over the last decades [31]. An increase in the incidence rate of low-grade NENs was also observed at various anatomical sites (lung, rectum, stomach, and appendix), whereas for others it remained stable (pancreas and small intestine) or decreased (colon). However, when comparing the trends overall, the incidence increase observed for high-grade NENs was only slightly higher, and substantially in the same order of magnitude to that observed for low-grade (high-grade 4.1-fold increase vs low-grade 3.2).

Our data are the first to report an incremental incidence trend for digestive high-grade NENs. Very few epidemiological data are available for this highly aggressive neuroendocrine cancer group, often excluded from epidemiology investigation on NENs [7, 32]. This incremental trend was consistently observed at all digestive sites investigated, grossly paralleling that observed for low-grade NENs.

Our findings raise the hypothesis that NENs share susceptibility factors independently of cancer grade, a phenomenon observed for other cancer types. As an example, in the upper and lower airways, smoking habits associate with various carcinomas, no matter the histological type and the cancer grade [33, 34]. High-grade cancer associates with severe genetic somatic abnormalities, usually involving genes controlling key cell proliferation pathways, and this is true for NENs too [35]. As for NENs, it could be hypothesized that the same, yet unknown factor(s) promote neuroendocrine carcinogenesis, and may then result in grade differences depending on the adding-on of key genetic or epigenetic alterations (either synchronous or metachronous).

As for gender and ethnicity, the general picture emerging from our data is that the patient with low-grade NENs is more frequently African–American of either sex, though prevalently female in case of NEN from the lung or the stomach. Similarly, high-grade NENs were more frequently observed in male African Americans, with the notable exception of the lung (and at lesser extent the appendix) for which NENs were predominantly observed in male white patients. So, male gender and African American ethnicity appear to play a role in determining NEN development risk.

Finally, the incidence of NEN in the 2012 in 18 SEER registries was 14.02 per 100,000, low-grade NEN being approximately 25% of all, and up to 57% when the high-grade NEN of the lung were excluded. In US, a cancer is rare for an incidence lower than 15 in 100,000 people; while in EU, a cancer is rare when lower than six in 100,000 people [36–38]. Our data suggest that NEN are close to outgrow the current US categorization, and well above the EU definition. In reality, when the high-grade NENs of the lung are excluded, NEN incidence is considerably lower (6.55 per 100,000) and far below the US cut-off definition for rare cancer, but still higher than the EU definition. A homogenous worldwide definition of rare cancer is probably needed, at least to uniform data analysis and to promote common cancer-specific policies.

Though we aimed at being as accurate as possible, some methodological limits are present. In specific, different morphological codes were used to classify NENs, and different standard populations were used to adjust for differences between countries in the age structure of the various populations. So, comparisons among different geographical areas or populations were subject to some bias. In addition, SEER data refer to tumors of proven malignancy only, and since “carcinoids” for long have been considered benign, this may have hampered data collection, especially between 1973–2000. Despite such limits, this study is based on data from 11 countries with a total population of over 100 million. Also, SEER was used as referral database since containing information on over 7.5 million cancer cases, providing the large number of events required for reliable estimation of incidence in rare cancers as NENs.

In conclusion, our data indicate that NENs overall are stably increasing independently of grade. This yet poorly understood phenomenon would require a major investigation effort to answer the expected rising demand for cure of the increased NEN cancer patients, as well as prevention of this group of neoplasms.

## Electronic supplementary material

Supplementary Information
