# Supplementary material for: Increased incidence trend of low-grade and high-grade neuroendocrine neoplasms
Source: Endocrine. 2017 Mar 16;58(2):368–79. doi: 10.1007/s12020-017-1273-x (PMC5671554; doi:10.1007/s12020-017-1273-x)
Supplement: Supplementary file 1 — Supplementary Information [file 12020_2017_1273_MOESM1_ESM.docx]

**Supplementary Figure 1 - Incidence of low-grade neuroendocrine neoplasms (NEN) according to gender in the United States, 2000–2012**

**
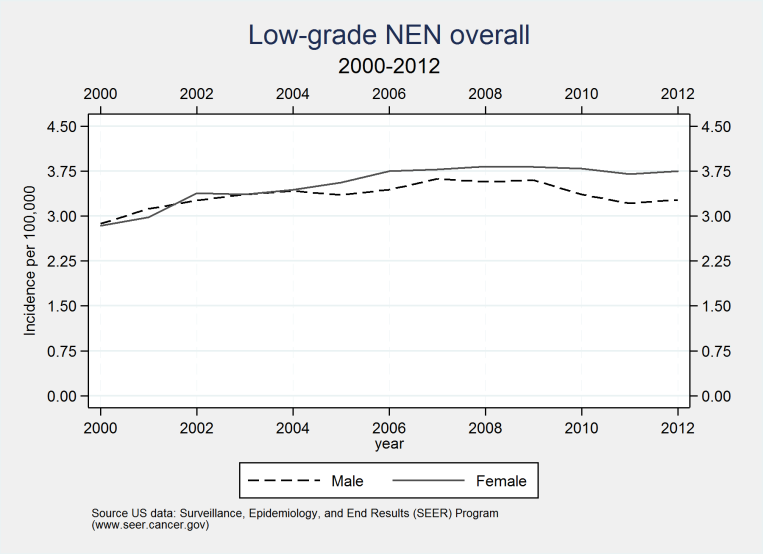

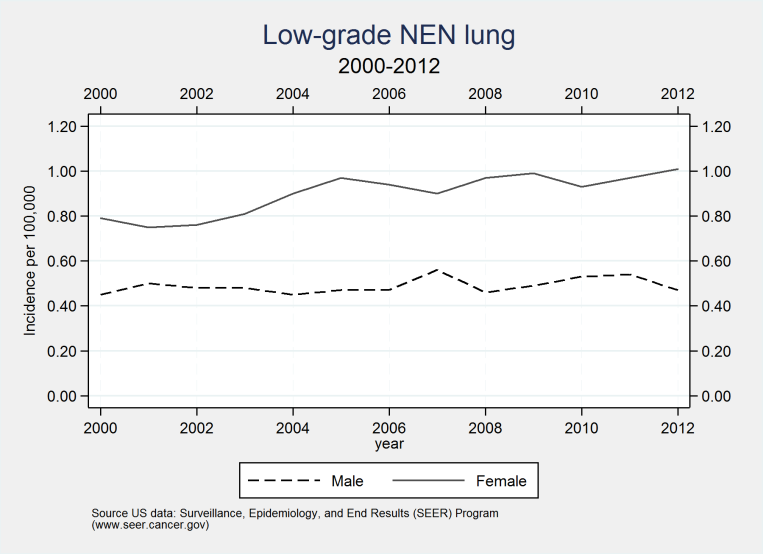
**

**
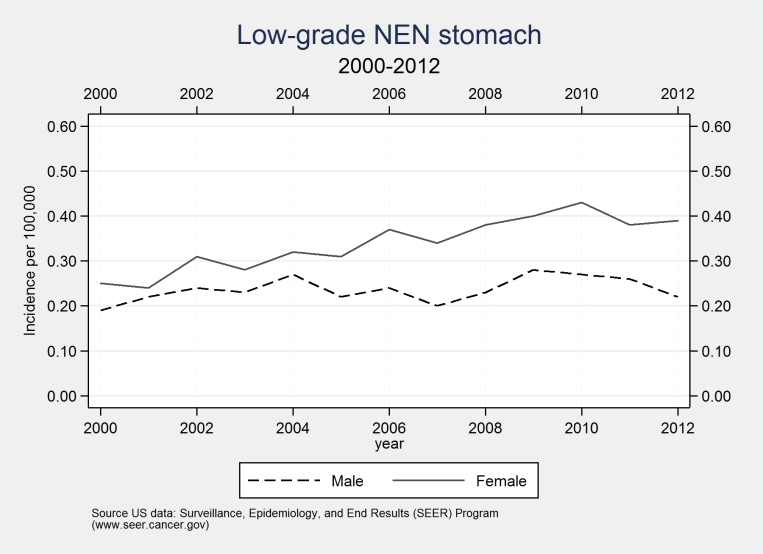

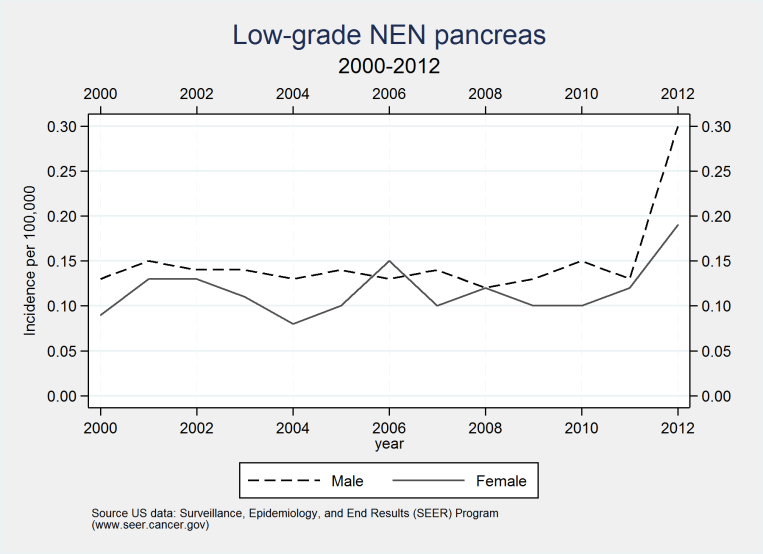

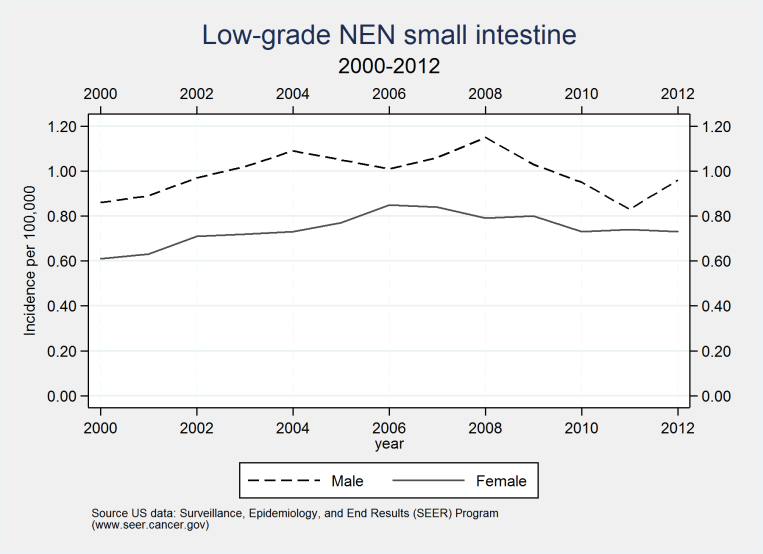

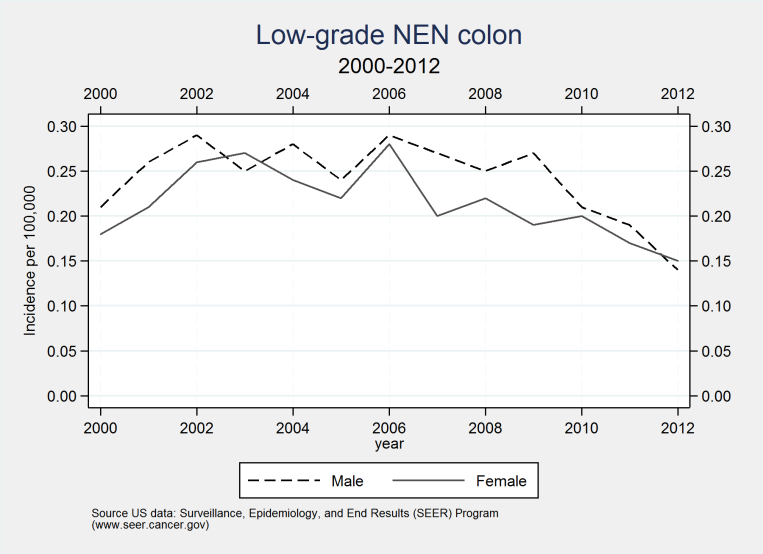
**

**
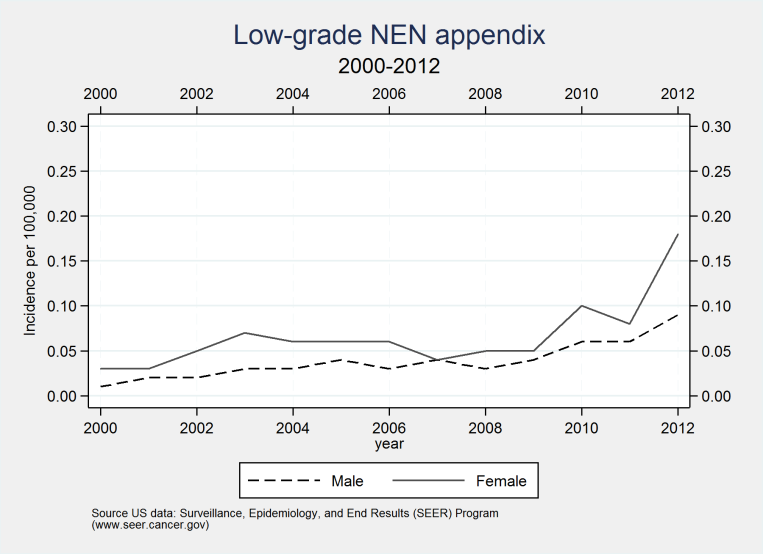

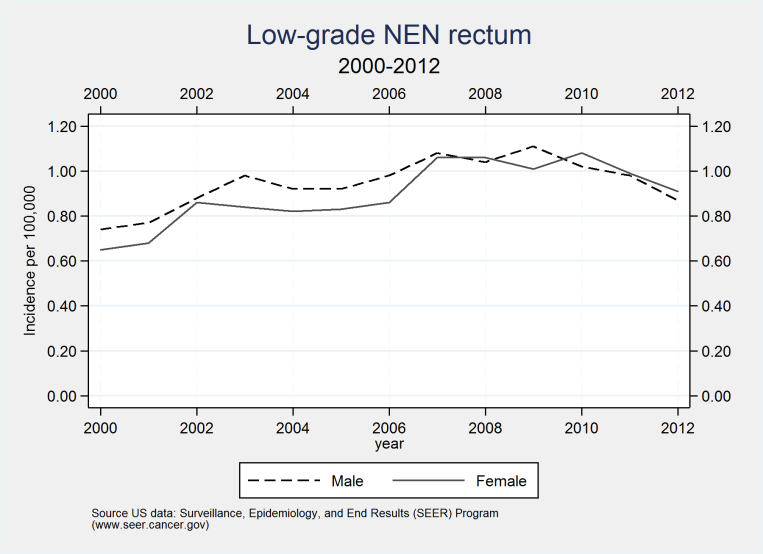
**

**
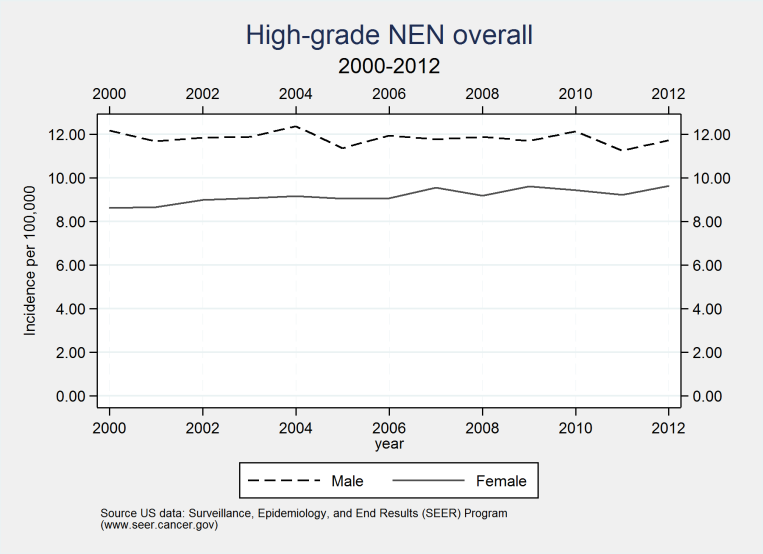
**

Source: Surveillance, Epidemiology, and End Results (SEER) Program (www.seer.cancer.gov)

**Supplementary Figure 2 - Incidence of high-grade neuroendocrine neoplasms (NEN) according to gender in the United States, 2000–2012**

**
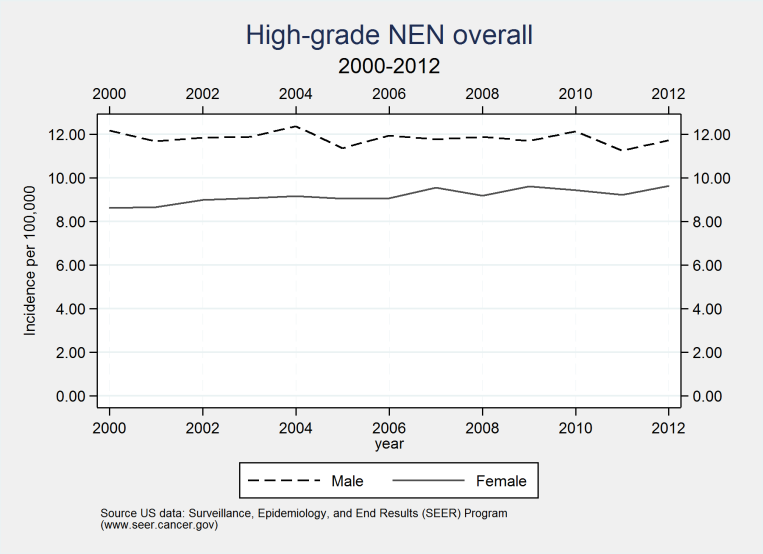

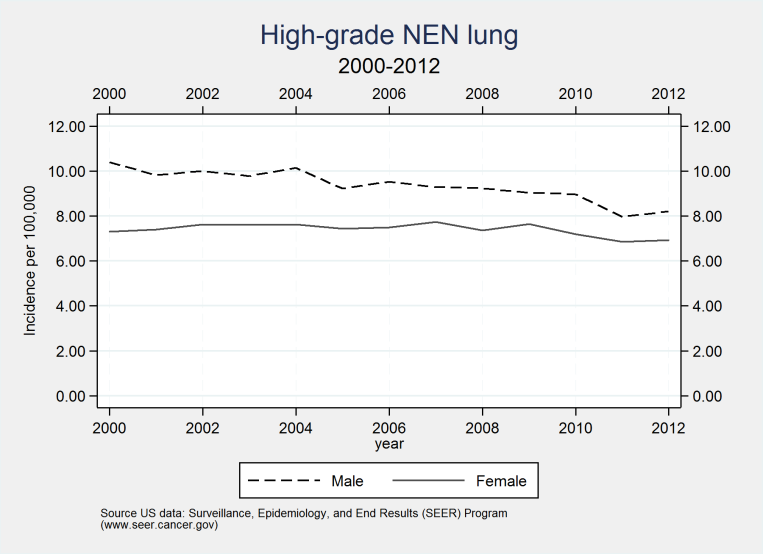
**

**
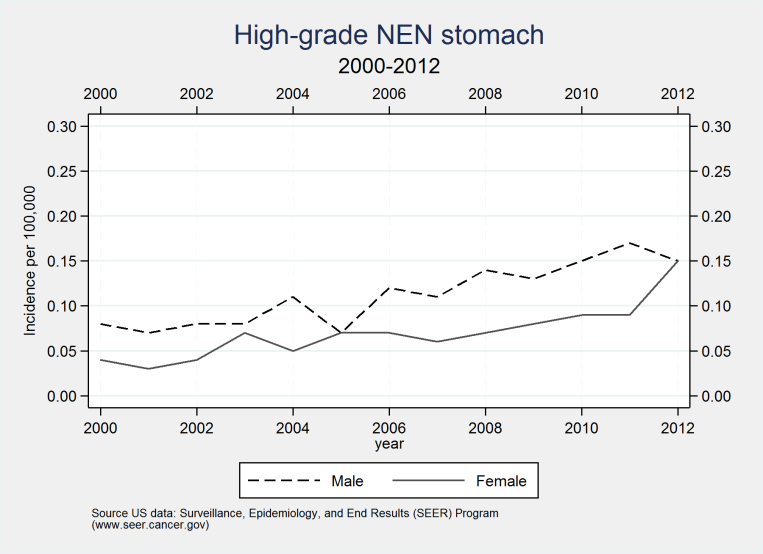

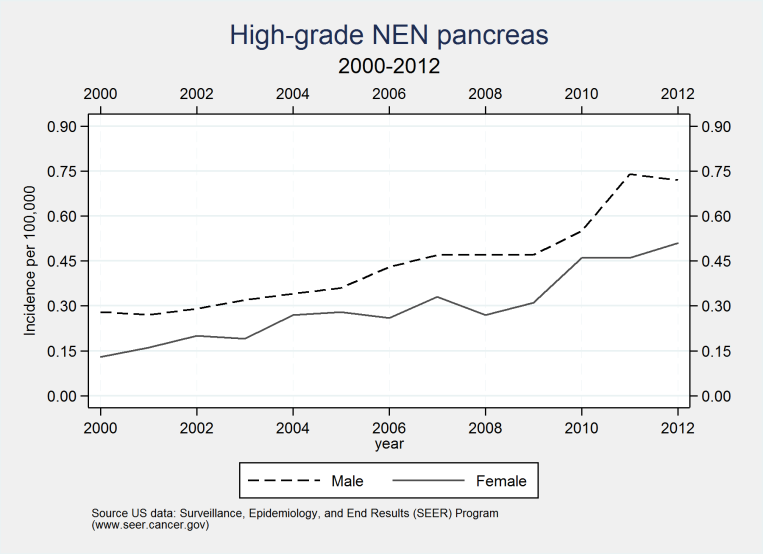

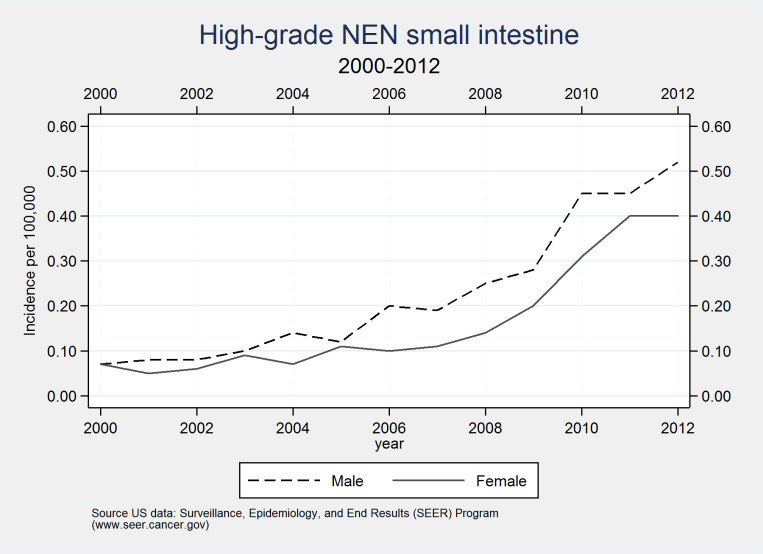

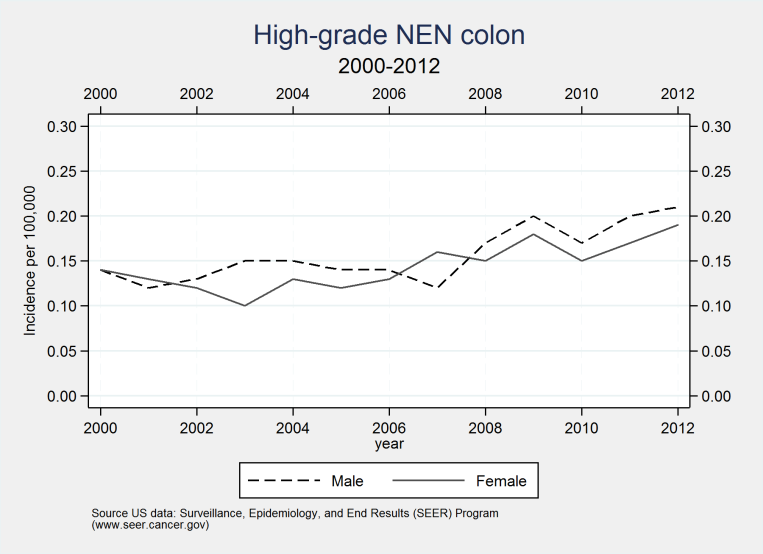
**

**
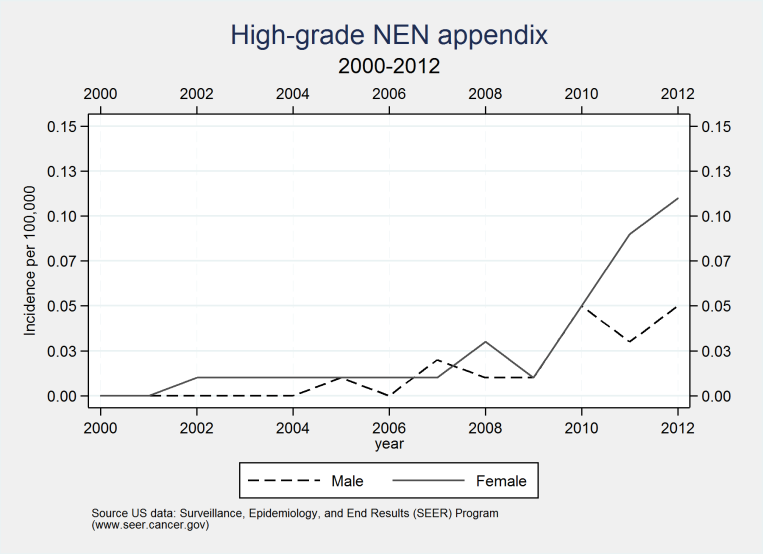

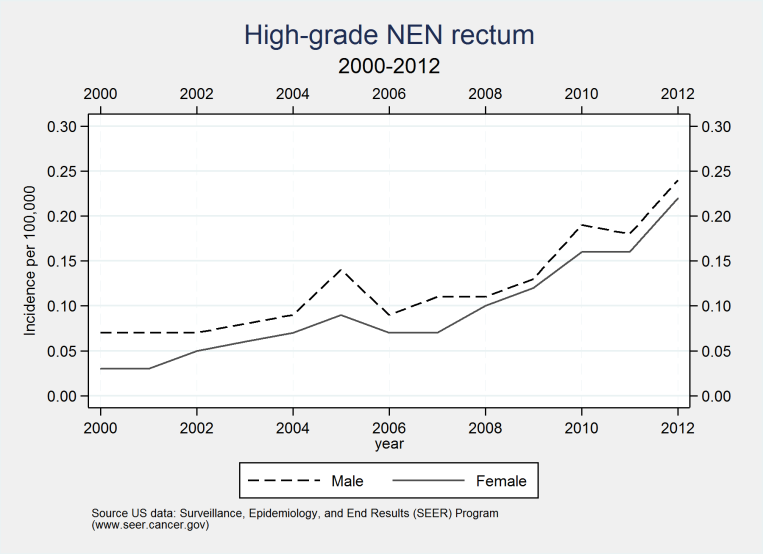
**

**
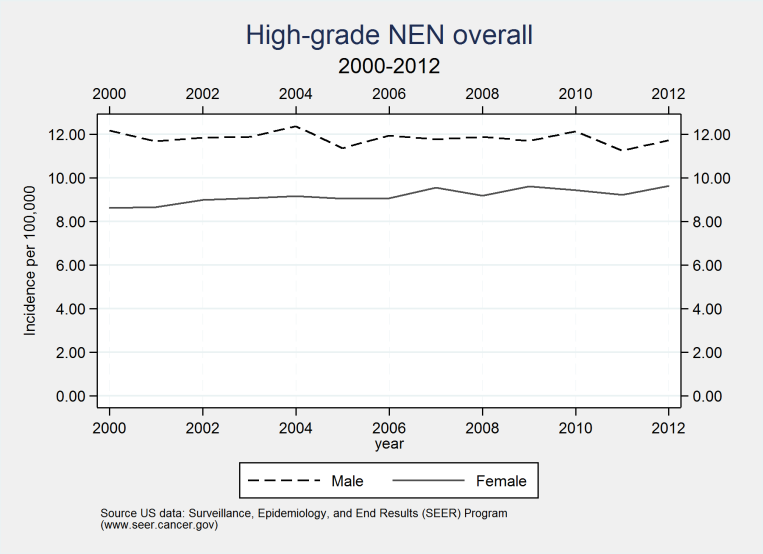
**

Source: Surveillance, Epidemiology, and End Results (SEER) Program (www.seer.cancer.gov)

**Supplementary Figure 3 - Incidence of low-grade neuroendocrine neoplasms (NEN) according to ethnicity in the United States, 2000–2012**

**
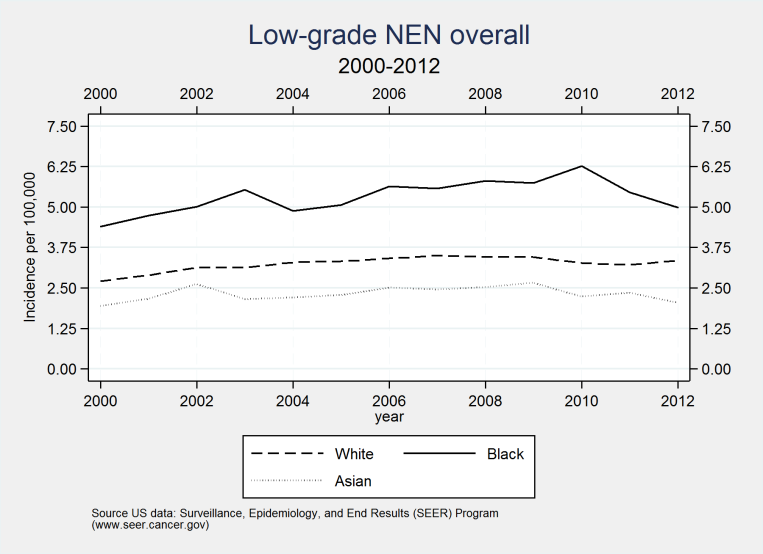

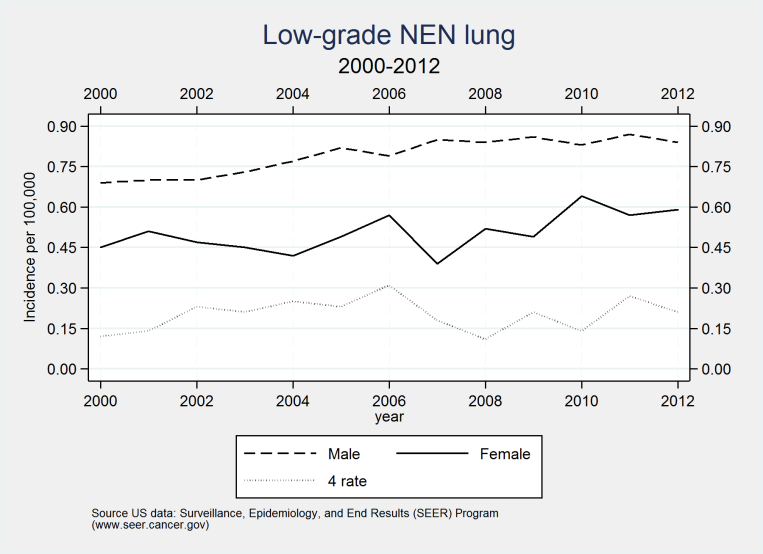
**

**
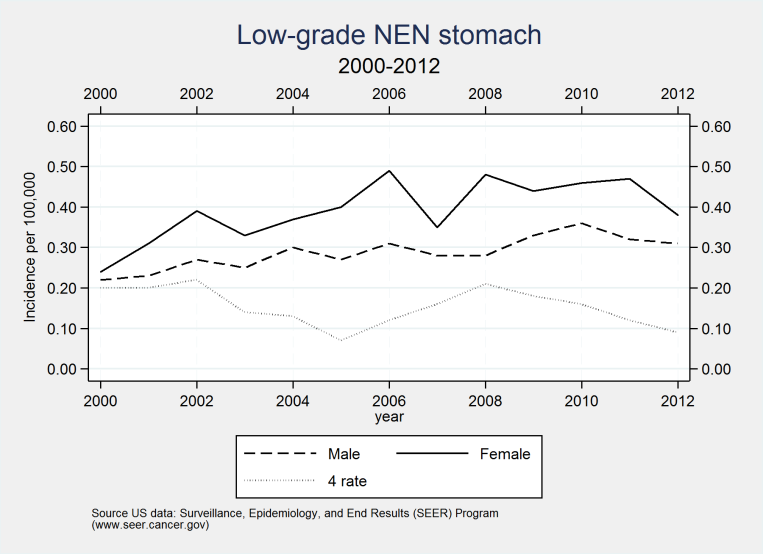

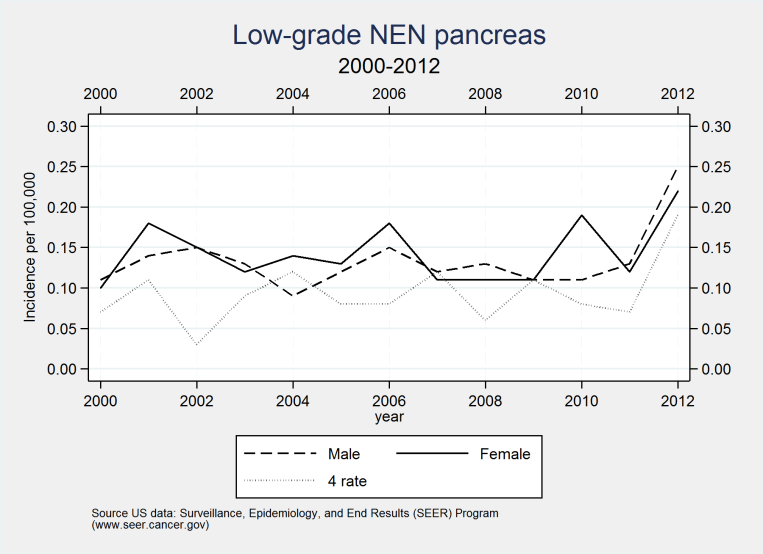

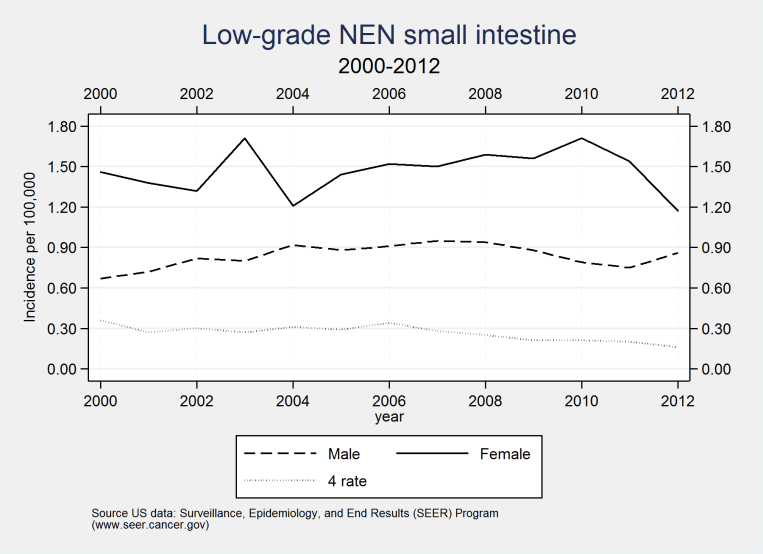

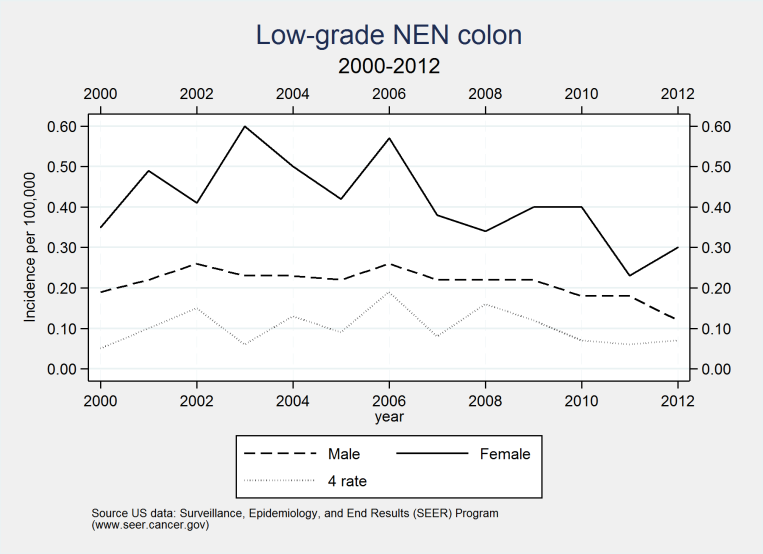
**

**
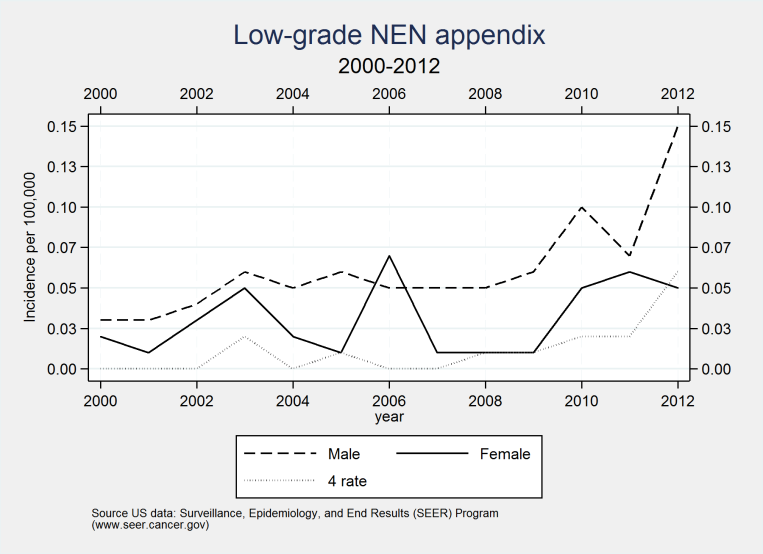

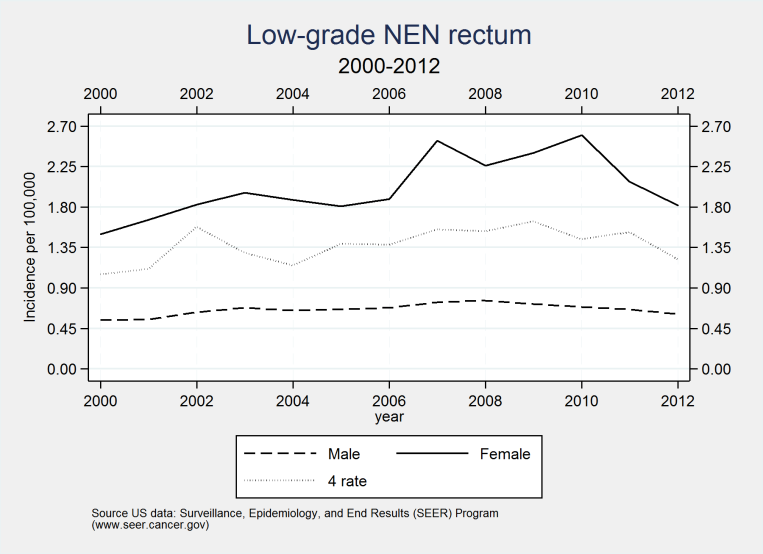
**

**
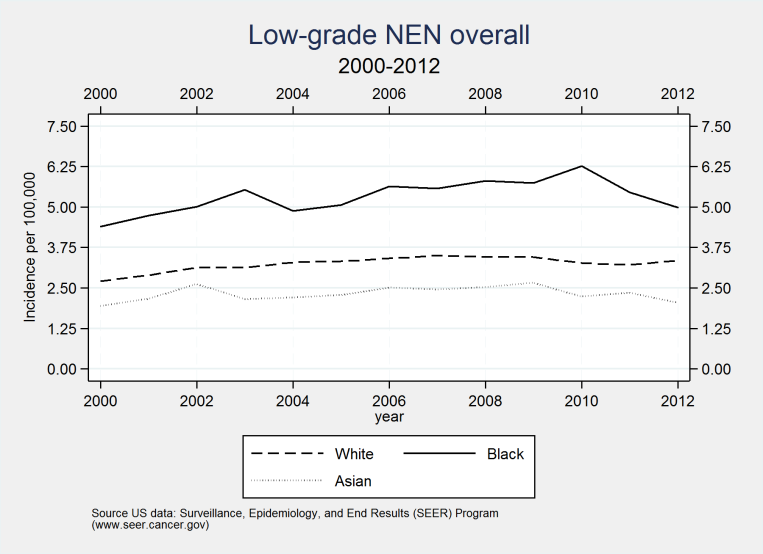
**

Source: Surveillance, Epidemiology, and End Results (SEER) Program (www.seer.cancer.gov)

**Supplementary Figure 4 - Incidence of high-grade neuroendocrine neoplasms (NEN) according to ethnicity in the United States, 2000–2012**

**
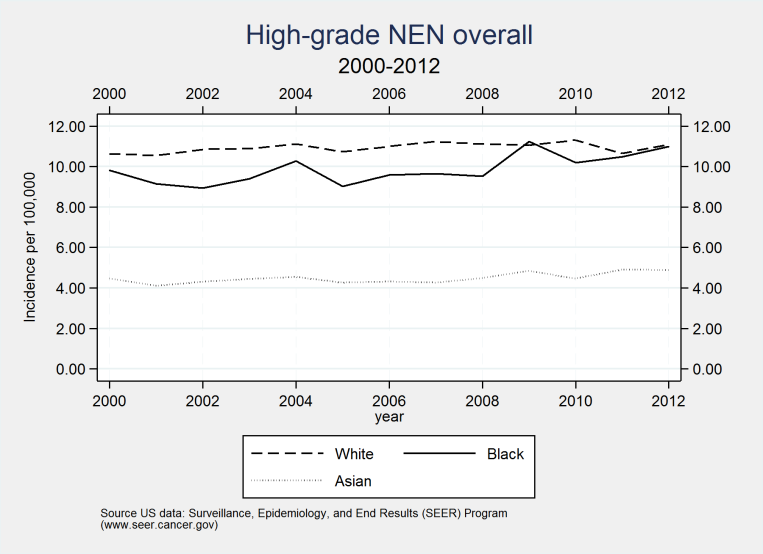

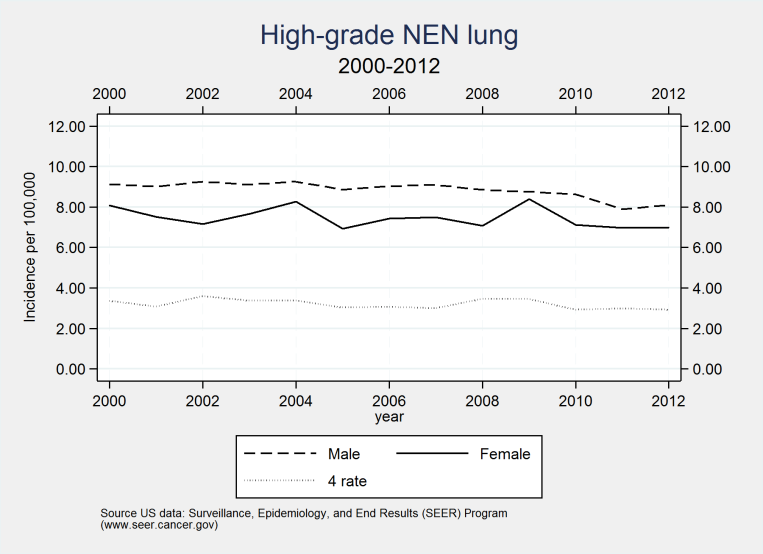
**

**
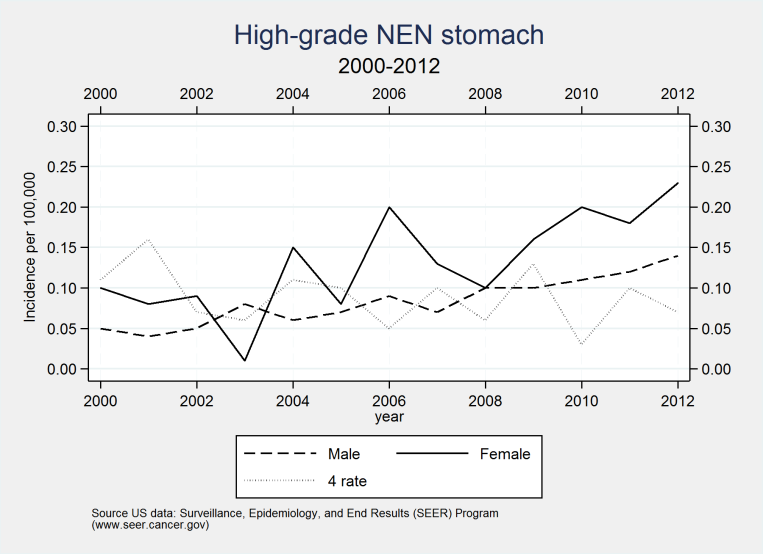

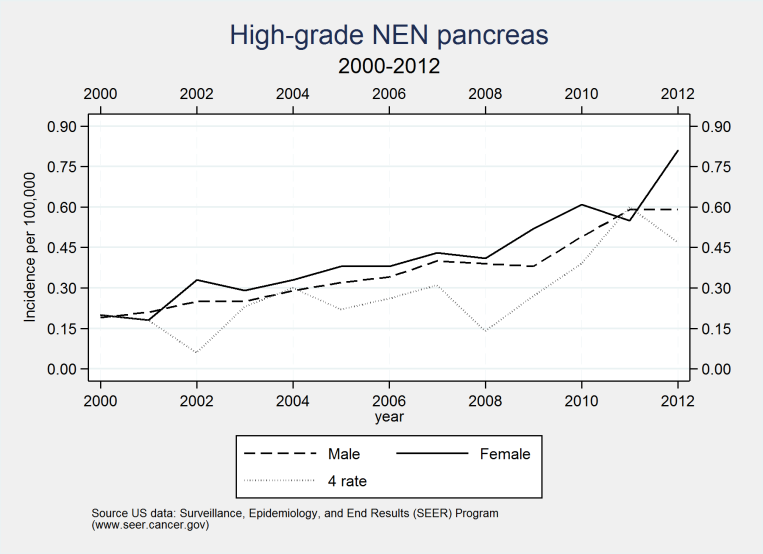

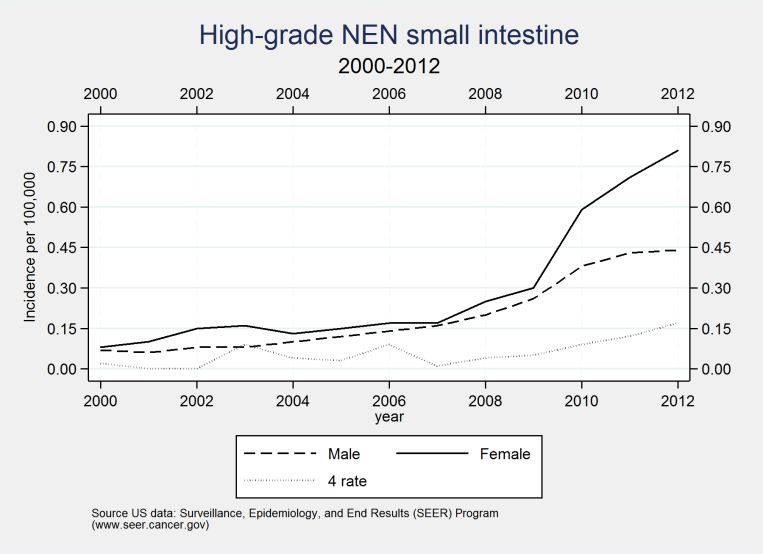

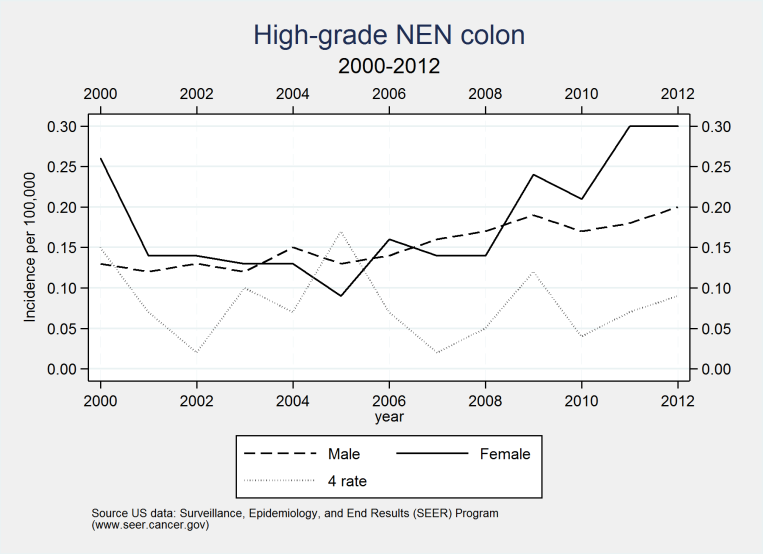
**

**
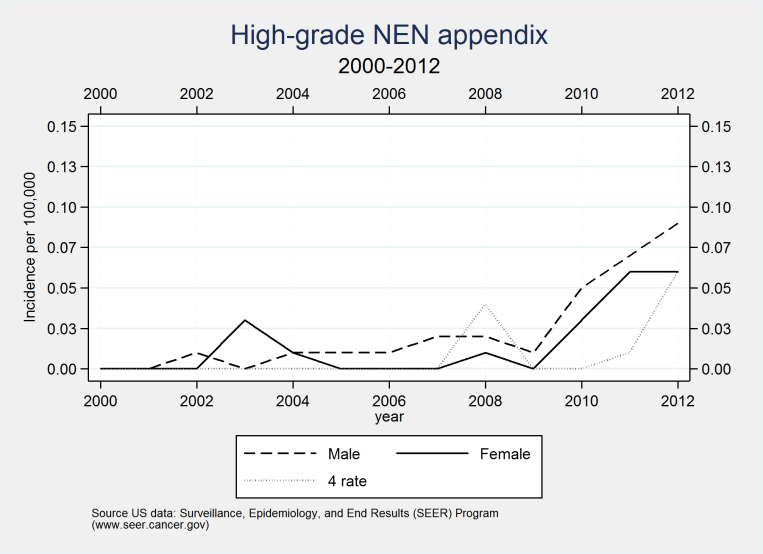

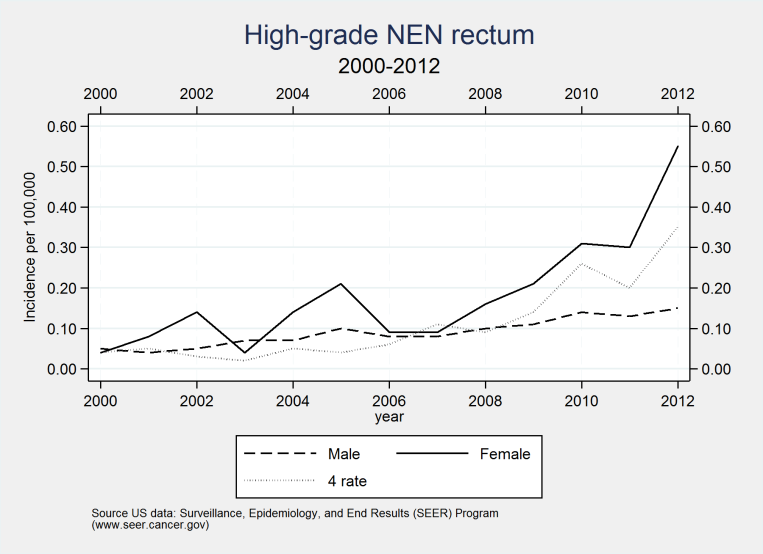
**

**
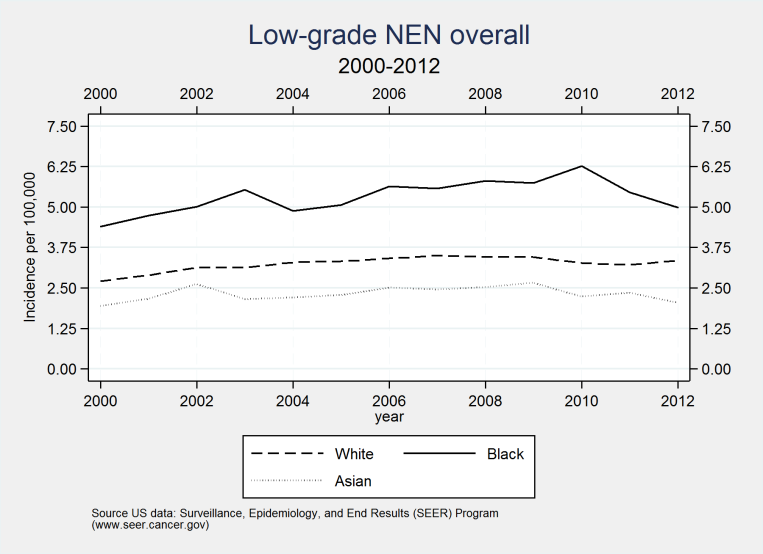
**

Source: Surveillance, Epidemiology, and End Results (SEER) Program (www.seer.cancer.gov)
